# CpG Island Mapping by Epigenome Prediction

**DOI:** 10.1371/journal.pcbi.0030110

**Published:** 2007-06-08

**Authors:** Christoph Bock, Jörn Walter, Martina Paulsen, Thomas Lengauer

**Affiliations:** 1 Max-Planck-Institut für Informatik, Saarbrücken, Germany; 2 Genetik/Epigenetik, Universität des Saarlandes, Saarbrücken, Germany; University of Massachusetts Medical School, United States of America

## Abstract

CpG islands were originally identified by epigenetic and functional properties, namely, absence of DNA methylation and frequent promoter association. However, this concept was quickly replaced by simple DNA sequence criteria, which allowed for genome-wide annotation of CpG islands in the absence of large-scale epigenetic datasets. Although widely used, the current CpG island criteria incur significant disadvantages: (1) reliance on arbitrary threshold parameters that bear little biological justification, (2) failure to account for widespread heterogeneity among CpG islands, and (3) apparent lack of specificity when applied to the human genome. This study is driven by the idea that a quantitative score of “CpG island strength” that incorporates epigenetic and functional aspects can help resolve these issues. We construct an epigenome prediction pipeline that links the DNA sequence of CpG islands to their epigenetic states, including DNA methylation, histone modifications, and chromatin accessibility. By training support vector machines on epigenetic data for CpG islands on human Chromosomes 21 and 22, we identify informative DNA attributes that correlate with open versus compact chromatin structures. These DNA attributes are used to predict the epigenetic states of all CpG islands genome-wide. Combining predictions for multiple epigenetic features, we estimate the inherent CpG island strength for each CpG island in the human genome, i.e., its inherent tendency to exhibit an open and transcriptionally competent chromatin structure. We extensively validate our results on independent datasets, showing that the CpG island strength predictions are applicable and informative across different tissues and cell types, and we derive improved maps of predicted “bona fide” CpG islands. The mapping of CpG islands by epigenome prediction is conceptually superior to identifying CpG islands by widely used sequence criteria since it links CpG island detection to their characteristic epigenetic and functional states. And it is superior to purely experimental epigenome mapping for CpG island detection since it abstracts from specific properties that are limited to a single cell type or tissue. In addition, using computational epigenetics methods we could identify high correlation between the epigenome and characteristics of the DNA sequence, a finding which emphasizes the need for a better understanding of the mechanistic links between genome and epigenome.

## Author Summary

A key challenge for bioinformatic research is the identification of regulatory regions in the human genome. Regulatory regions are DNA elements that control gene expression and thereby contribute to the organism's phenotype. An important class of regulatory regions consists of so-called CpG islands, which are characterized by frequent occurrence of the CG sequence pattern. CpG islands are strongly associated with open and transcriptionally competent chromatin structure, they play a critical role in gene regulation, and they are involved in the epigenetic causes of cancer. In this article we make several conceptual improvements to the definition and mapping of CpG islands. First, we show that the traditional distinction between CpG islands and non-CpG islands is too harsh, and instead we propose a quantitative measure of CpG island strength to gradually distinguish between stronger and weaker regulatory regions. Second, by genome-wide comparison of multiple epigenome datasets we identify high correlation between features of the genome's DNA sequence and the epigenome, indicating strong functional interdependence. Third, we develop and apply a novel method for predicting the strength of all CpG islands in the human genome, giving rise to an improved and more accurate CpG island mapping.

## Introduction

CpG islands are genomic regions characterized by an exceptionally high CpG dinucleotide frequency [1–3]. In humans, they are among the most important regulatory regions, with functional roles in both normal and disease-related gene expression [4,5].

Originally, CpG islands were discovered by virtue of an epigenetic property, namely, the absence of DNA methylation: when the human genome was experimentally digested with methylation-sensitive restriction enzymes, some genomic regions were cut into small fragments, while the bulk of the genome remained uncut [6]. Since the restriction enzyme (HpaII) used cuts DNA only at unmethylated CpG dinucleotides, it was concluded that a small but significant fraction of the genome is reproducibly unmethylated.

After a sample of these so-called HpaII tiny fragments had been sequenced, it became obvious that they were highly GC-rich and CpG-rich [3]. In an early computational analysis, this observation was utilized to define such regions as CpG islands, and a simple set of criteria was suggested to identify them based on their DNA sequence alone [7]. According to these so-called Gardiner-Garden sequence criteria, a genomic region has to fulfill three conditions to classify as a CpG island: (1) GC content above 50%, (2) ratio of observed-to-expected number of CpG dinucleotides above 0.6, and (3) length greater than 200 basepairs (bp). Because the amount of sequence data strongly outnumbered the experimental data available for DNA methylation, this definition quickly replaced the original methylation-based concept.

Since their initial discovery, CpG islands have been subject to extensive research. Today it is known that CpG islands (according to the DNA sequence criteria mentioned above or slightly modified variants) associate with more than three-quarters of all known transcription start sites [8] and with 88% of active promoters identified in primary fibroblasts [9], indicating that they bear important regulatory functions. Furthermore, they are hot spots of specific histone modifications [10,11], they frequently bind ubiquitous transcription factors such as SP1 [12], and they exhibit particularly accessible chromatin structures [13]. For these reasons, CpG islands have become indispensable for genome analysis and annotation. For example, they play a fundamental role for promoter prediction [14], and their use as candidate regions for aberrant DNA methylation has contributed significantly to our understanding of the epigenetic causes of cancer [15].

However, the current sequence-based definitions of CpG islands [7,16] incur several disadvantages, which hamper both their theoretical and practical value. First, they are based on three threshold parameters that lack a clear biological justification. For example, it is unclear why 200 bp should be the most appropriate minimum length to define CpG islands, especially since even a random permutation of the genome sequence would give rise to a substantial number of CpG islands with this minimum length. A length threshold of 500 bp is also widely used, and its use was motivated by its ability to exclude most Alu-repeat-associated regions [16], but again, no systematic analysis or parameter selection method has been applied to justify this particular value.

Second, current definitions are purely binary, i.e., a particular region either qualifies as a CpG island or not. This not only fails to account for the fact that CpG islands can differ considerably in terms of their sequence composition and epigenetic states [17], it can also lead to unintuitive special cases. For example, even if a short CpG-rich region fails to fulfill CpG island criteria on its own, the same region may well fulfill the criteria after small and seemingly unrelated changes of a few neighboring nucleotides. Thus, the mapping of CpG islands is inherently unstable and depends not only on the definition used but also on the exact implementation of the mapping software. In contrast, the introduction of a numerical score for CpG island strength would allow distinguishing weak, intermediate, and strong CpG islands, without the necessity of a fixed all-or-nothing threshold.

Third, and most critically, sequence-based CpG island criteria fail to distinguish between “bona fide” CpG islands—which are typically unmethylated, serve as transcription regulators, and exhibit an open and transcriptionally competent chromatin structure—and CpG-rich regions lacking these characteristics. More precisely, current CpG island criteria seem to be sufficiently sensitive in the sense that they detect most bona fide CpG islands in the human genome, but their specificity is low, i.e., they give rise to a substantial number of false positive classifications. For example, Yamada et al. observed that almost a third of the putative CpG islands analyzed showed significant DNA methylation [18], in contradiction to the original concept of CpG islands as unmethylated regions.

To resolve the significant drawbacks of current sequence-based CpG island criteria, it was suggested to abandon the concept of CpG islands altogether and to replace it by direct counting of CpG dinucleotides [19]. In this study, we propose a less radical but arguably more viable strategy. Our approach maintains the high sensitivity of current CpG island criteria, but substantially improves their specificity, it introduces a more biologically meaningful way of selecting thresholds, and it accounts for the fact that CpG islands quantitatively differ in their strength.

The fundamental concept of this study is to combine an initial, sequence-based mapping of CpG islands with subsequent prediction of CpG island strength. CpG island strength is expressed as a single quantitative score per CpG island, summarizing its inherent tendency—across different cell types and tissues—to exhibit an unmethylated, open, and transcriptionally competent chromatin structure. It is calculated as a combination of epigenome predictions and provides a measure for discrimination between bona fide CpG islands and regions that are just CpG-rich but show no evidence of the epigenetic and functional characteristics of bona fide CpG islands. We evaluate the predicted CpG island scores by comparison with large-scale experimental datasets on DNA methylation and transcription initiation sites, since absence of DNA methylation and presence of promoter activity are regarded as characteristic of bona fide CpG islands.

**Figure 1:**
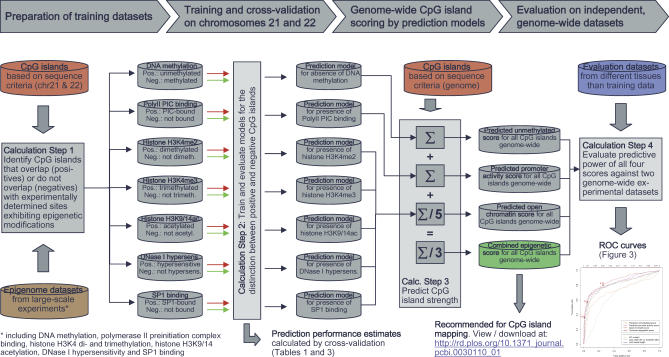
Conceptual Overview This figure outlines the workflow used in this study to derive quantitative scores of CpG island strength, and to evaluate their performance as predictors of bona fide CpG islands. The arrows at the top describe the phases of the analysis, the cylinders correspond to input datasets (orange, blue, and brown cylinders) and results datasets (grey and green cylinders), and the rectangular boxes represent major computational steps. The sigmas in the calculation step 3 box stand for summation over the input. The figure is slightly simplified and focuses on a single CpG island map. In fact, the entire workflow was performed separately for three CpG island maps that differ in the repeat-exclusion strategy used (TJU, GGF, and GGM), with subsequent benchmarking of their performances (Figure 5).

Figure 1 provides a schematic overview of our approach, which is necessarily complex since we derive and benchmark four different scores of CpG island strength using combinations of large-scale epigenome datasets. From left to right, the first step comprises preparation of seven training datasets, based on pairwise overlaps between CpG island maps and epigenome datasets. In the second step, a prediction model is trained and its performance is estimated for each training dataset. The resulting prediction models are then used to score all CpG islands genome-wide. From these scores—in step three—four CpG island scores are calculated. In step four, a performance comparison on two large-scale evaluation datasets shows that the “combined epigenetic score” is the best indicator of CpG island strength and most predictive of bona fide CpG islands. All training and testing in this study is performed on Chromosomes 21 and 22 for reasons of data availability. Predictions are calculated and validated on the entire genome. The entire workflow as outlined in Figure 1 was repeated three times, for three widely used CpG island maps. By comparing the results, we show that CpG island strength predictions provide an improvement over each map, and we are able to select the most appropriate setup for the final maps of predicted bona fide CpG islands.

## Results

### Preparation of Traditional CpG Island Maps As the Basis for Prediction

Our prediction of CpG island strength and mapping of bona fide CpG islands started from traditional CpG island maps, which we derived by means of widely used sequence-based CpG island criteria. This approach is unlikely to significantly reduce the completeness of our mapping since the original CpG island criteria [7] are regarded as highly sensitive and there is no evidence that they miss a substantial number of bona fide CpG islands.

The application of traditional CpG island finder algorithms faces the problem of repetitive DNA in the genome. Some evolutionarily recent repeat insertions are CpG-rich (e.g., Alu elements) and could erroneously be identified as CpG islands even though they most likely bear little regulatory function [16]. Several methods have been suggested to address this problem, but their efficacy has not been systematically investigated. We therefore applied and compared three widely used calculation methods: (1) repeat exclusion by using strict thresholds for GC content (55%), CpG observed-to-expected ratio (0.65), and CpG island length (500 bp) as suggested by Takai and Jones [16]; (2) repeat exclusion by combining the standard Gardiner-Garden thresholds [7] with subsequent removal of all CpG islands that comprise less than 200 bp of nonrepetitive DNA; and (3) repeat exclusion by applying the standard thresholds [7] to the repeat-masked genome.

Using each of these methods, we derived a genome-wide map of CpG islands. Method 1, which we refer to as TJU (for “Takai Jones unmasked”), gave rise to 37,531 CpG islands genome-wide. Method 2, which we refer to as GGF (for “Gardiner-Garden filtered”), gave rise to 94,450 CpG islands genome-wide. And method 3, which we refer to as GGM (“Gardiner-Garden masked”), gave rise to 109,600 CpG islands genome-wide. All three maps were processed in parallel through most of this study.

### Establishment of Training Datasets for CpG Islands Strength Prediction

Absence of DNA methylation and presence of promoter activity are regarded as characteristic of bona fide CpG islands. Therefore, we hypothesized that computational predictions of DNA methylation and promoter activity might provide suitable scores of CpG island strength and thus indicators for the genome-wide mapping of bona fide CpG islands. In previous work focusing on human lymphocytes, we showed that prediction of CpG island methylation is possible with high accuracy based on the DNA sequence plus additional information such as the DNA helix structure and the distribution of repetitive DNA elements [20]. Our finding has recently been independently confirmed for brain tissue [21,22] and is expected to hold for a wide range of cell types and tissues. Computational promoter prediction is a well-studied topic and is also feasible with high accuracy across different cell types and tissues (see Bajic et al. [14] and references therein).

We therefore prepared training datasets for DNA methylation and promoter activity (calculation step 1 in Figure 1), to be processed with our epigenome prediction pipeline (see next section). Each training dataset was constructed by identifying pairwise overlaps between the three CpG island maps (Figure 1, orange cylinder) and experimental epigenome datasets on DNA methylation and promoter activity (Figure 1, brown cylinder), giving rise to a set of positives (i.e., regions that exhibit characteristics of bona fide CpG islands) as well as a set of negatives (i.e., regions that do not) for both DNA methylation and promoter activity (Figure 1, grey cylinders between calculation steps 1 and 2). For the prediction of unmethylated versus methylated CpG islands, training datasets were constructed using DNA methylation data that Yamada et al. established for Chromosome 21q [18]. Similarly, for the prediction of CpG islands that show evidence of promoter activity versus those that do not, training datasets were constructed using the genome-wide list of polymerase II preinitiation complex binding sites that Kim et al. established for primary fibroblasts [9] (for consistency with additional predictions that we report below, we restricted the latter dataset to Chromosomes 21 and 22).

### Construction of a General Pipeline for Epigenome Prediction

Based on our experience with DNA methylation prediction [20], we constructed a general epigenome prediction pipeline, which performs calculation step 2 in the overview diagram (Figure 1). Each prediction takes a training dataset as input and performs three subsequent steps: calculation of prediction attributes, performance estimation by cross-validation, and genome-wide prediction. The outputs of the pipeline are an overall performance estimate, a table of most predictive attributes, and a predicted score for each CpG island genome-wide.

More specifically, these steps are performed as follows. (1) Prediction attributes are calculated: for each case in the respective training dataset, the pipeline calculates 847 potentially predictive attributes from genome data. These attributes belong to six groups: DNA sequence patterns, repeat distribution, predicted DNA helix structure [23,24], predicted transcription factor binding sites, genetic variation, and CpG island attributes (genes and gene-related information are deliberately omitted to ensure that the predictions are independent of manual curation expertise). (2) Performance is estimated by cross-validation: using the above attributes and the training data, the pipeline trains a linear support vector machine to predict whether a CpG island belongs to the set of positives or to the set of negatives. Prediction performance is evaluated by calculating the average correlation and accuracy over ten runs of a 10-fold cross-validation. Furthermore, to understand which attributes contribute most significantly to high prediction performance, two additional analyses are performed. First, the support vector machine is trained not only on the combination of all attributes but also on each of the six attribute groups separately. Second, Wilcoxon rank-sum tests are calculated to identify the most significant of all 847 attributes. On this basis, the optimal combination of attribute groups can be selected (we use repeat distribution plus predicted DNA helix structure plus CpG island attributes throughout this study because these three attribute groups achieve high prediction performance and capture complementary aspects of the DNA). (3) CpG island scores are predicted genome-wide: the linear support vector machine is trained on all training data and is then applied to calculate a prediction score between zero and one for all CpG islands genome-wide. The resulting score describes the likelihood that a particular CpG island belongs to the set of positives (i.e., regions that exhibit characteristics of bona fide CpG islands) and is therefore a potential indicator of CpG island strength.

### CpG Island Strength Estimated by Predicted DNA Methylation and Promoter Activity

Processing the training data for DNA methylation and promoter activity through our epigenome prediction pipeline showed that the pipeline can distinguish with high accuracy between unmethylated and methylated CpG islands and, similarly, between CpG islands that exhibit evidence of promoter activity (namely polymerase II preinitiation complex binding sites) and those that do not (Tables 1, S1, and S2). A closer inspection of the most predictive attributes helps us to understand how this prediction performance is achieved (Tables S3 and S4). First, unmethylated CpG islands contain significantly fewer tandem repeats and segmental duplications than their methylated counterparts. Second, polymerase II preinitiation complex–bound CpG islands overlap more frequently with highly conserved regions than do unbound CpG islands. And third, both unmethylated and polymerase II preinitiation complex–bound CpG islands are highly enriched with CpG-rich sequence patterns and regions of low predicted DNA rise (which is an important aspect of DNA helix structure [23,24]). These results support the hypothesis that the prediction score for DNA methylation at CpG islands as well as the prediction score for polymerase II preinitiation complex binding at CpG islands are both suitable indicators of CpG island strength. We denote their genome-wide prediction values derived by the epigenome prediction pipeline as the “predicted unmethylated score” and the “predicted promoter activity score,” respectively, and evaluate their predictiveness for CpG island strength below.

**Table 1:**
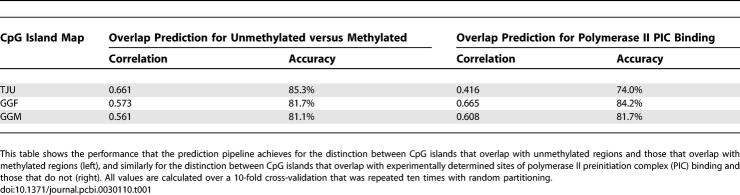
Prediction Performance for DNA Methylation and Promoter Activity at CpG Islands

### CpG Island Strength Estimated by Predicted Epigenetic State and Chromatin Structure

CpG island scores that focus exclusively on the absence of DNA methylation or on evidence of promoter activity may be insufficient for capturing all aspects of the complex epigenetic and functional states that characterizes bona fide CpG islands. To construct a more comprehensive epigenetic scoring of CpG island strength, we collected five additional large-scale epigenome datasets from the literature, each one describing a different aspect of an open and transcriptionally competent chromatin structure: histone H3K4 di- and trimethylation [10], histone H3K9/14 acetylation [10], DNase I hypersensitivity [13] and SP1 transcription factor binding [12]. All these datasets cover the nonrepetitive parts of human Chromosomes 21 and 22, to which we confine our analysis.

**Figure 2:**
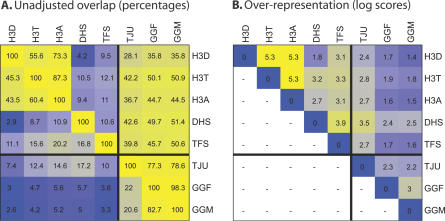
Co-Localization between the Five Components of the Open Chromatin Score and the Three CpG Island Maps (A) shows the relative frequency of overlap between epigenetically modified sites and CpG islands (percentage values). (B) shows the degree of over-representation relative to a simulated case where sites are uniformly distributed over the chromosomes (base-2 log scores). Yellow boxes correspond to frequent overlap, blue boxes to rare overlap. H3D, histone H3K4 dimethylation; H3T, histone H3K4 trimethylation; H3A, histone H3K9/14 acetylation; DHS, DNase I hypersensitive sites; TFS, SP1 transcription factor binding, plus the CpG island abbreviations used throughout this study (TJU, GGF, and GGM). (B) is symmetrical as the result of averaging, therefore only the upper right triangular matrix is reported. (A) is not symmetrical, as is obvious from an example: 51.4% of all 578 known DNase I hypersensitive sites on Chromosomes 21 and 22 overlap with a GGM CpG island, while only 5.0% of all 5,913 GGM CpG islands overlap with an experimentally determined DNase I hypersensitive site.

A genomic co-localization analysis that we performed showed that all five datasets of epigenetically modified regions indeed exhibit significant overlap with all three CpG island maps (Figure 2). Briefly, this analysis involved two steps. First, the absolute number of pairwise overlaps along Chromosomes 21 and 22 was counted for each pairwise combination of epigenetic modification map and CpG island map (Figure 2A). Second, these numbers were normalized by the expected frequency of overlap under the assumption of CpG islands and epigenetically modified regions being uniformly distributed (Figure 2B), to correct for length and frequency differences (see Materials and Methods for details).

Intriguingly, the enrichment observed in the genomic co-localization analysis was not independent between datasets but highly skewed towards a specific subset of CpG islands that frequently overlap with several epigenetic modifications simultaneously (Table 2). For example, CpG islands that show evidence of two out of five epigenetic modifications simultaneously are observed 10-fold to 20-fold more frequently than expected under a random model. We therefore concluded that all five epigenetic modifications do in fact capture different epigenetic indicators of a single concept, namely, whether or not a particular CpG island fosters an open and transcriptionally competent chromatin structure.

**Table 2:**
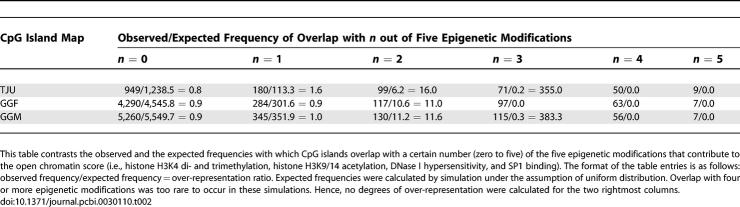
A Subset of CpG Islands Exhibits Highly Significant Overlap with Multiple Epigenetic Modifications Simultaneously

To convert this observation into a method for scoring CpG island strength, we prepared training datasets and applied our prediction pipeline separately for each of the five epigenetic modifications (calculation steps 1 and 2 in Figure 1). In all cases, the support vector machine was able to distinguish with significant accuracy between CpG islands that overlap with the particular epigenetic modification and those that do not (Tables 3 and S5). A closer inspection of the most predictive attributes showed that CpG islands exhibiting overlap with the epigenetic modification are more likely to contain CpG-rich patterns, are more conserved, and exhibit a characteristic predicted helix structure (see Table S6 for a list of most significant differences). Furthermore, we observed high correlations between the prediction scores for all five epigenetic modifications (Table S7), which provided additional support for the conclusion that they represent aspects of a single concept. Therefore, for each CpG island we calculated the average over all five predictions and thereby derived a single “open chromatin score” (calculation step 3 in Figure 1). Finally, since the predicted unmethylated score, the predicted promoter activity score, and the open chromatin score can be assumed to capture complementary aspects of a CpG island's epigenetic and functional state, we combined these three scores into an additional consensus score that we call the “combined epigenetic score” of CpG island strength.

**Table 3:**
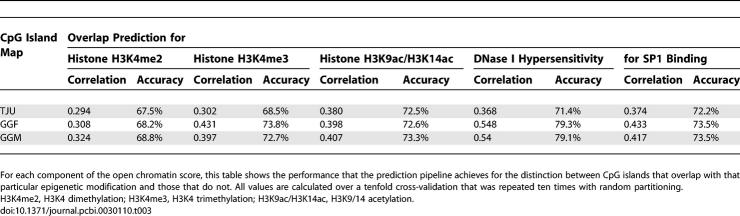
Prediction Performance for the Distinction between CpG Islands That Overlap with a Particular Epigenetic Modification and Those That Do Not

### Independent Evaluation of CpG Island Strength Predictions

#### 

For each of the predictions described above, the performance was assessed by means of cross-validation (Tables 1, 3, S1, S2, and S5). While this procedure can provide an accurate estimate of the prediction performance expected on new data of the same type, it is not sufficient for establishing the prediction scores as a quantitative indicator of CpG island strength. First, all training and testing was restricted to Chromosomes 21 and 22; therefore, it could not be assessed how well the predictions generalize to the entire genome. Second, cross-validation on a single dataset cannot exclude the risk of overfitting to the special properties of this particular dataset, which can include both biological factors (such as tissue-specific and cell-type-specific effects) and experimental problems (such as bias towards specific genome regions).

Therefore, we performed an additional evaluation, based on two large-scale datasets (Figure 1, blue cylinder): (1) a random sample of unmethylated and methylated regions in the human genome derived from brain tissue by means of large-scale tag sequencing of DNA fragments generated by methylation-sensitive restriction enzymes [25], and (2) a genome-wide map of experimentally determined transcription start sites obtained for a wide range of tissues by the FANTOM3 project [26]. Independent evaluation (without retraining) on these datasets can overcome both limitations of the previously described cross-validations. First, the two datasets cover the entire (nonrepetitive) human genome, not only two chromosomes like the training data. Second, both datasets deviate significantly in terms of tissue type, cell type, and experimental protocol from all training datasets used throughout this study. Hence, any significant prediction performance that the CpG island scores achieve on these evaluation datasets can be attributed to inherent and robust properties of the CpG islands themselves.

The first evaluation dataset was constructed by identifying overlap between CpG islands and regions of known methylation state, giving rise to experimentally positive CpG islands (i.e., overlapping with unmethylated regions) and experimentally negative CpG islands (i.e., overlapping with methylated regions). The second evaluation dataset was constructed by identifying overlap between CpG islands and experimentally determined transcription start sites. CpG islands that harbor at least three independent transcription initiation events were included in the set of positives, while all remaining CpG islands were included in the set of negatives.

All four CpG island scores were then evaluated against these two evaluation datasets using receiver operating characteristic (ROC) curves, which is the standard method for benchmarking classifiers in machine learning [27] (calculation step 4 in Figure 1). These ROC curves interpret the score of any one CpG island as its predicted likelihood of being a bona fide CpG island. For all possible thresholds on the CpG island score, they describe the tradeoff between the true positive rate (i.e., the percentage of bona fide CpG islands that are detected, also called sensitivity) and the false positive rate (i.e., the percentage of negatives that are erroneously classified as bona fide CpG islands, which is equal to one minus specificity) and thereby assess how well the particular CpG island score predicts the evaluation datasets. A purely random score would on average result in a ROC curve that is a straight line from (0,0) to (1,1); the closer the curve bends towards the top left corner, the better is the performance of the evaluated CpG island score.

**Figure 3:**
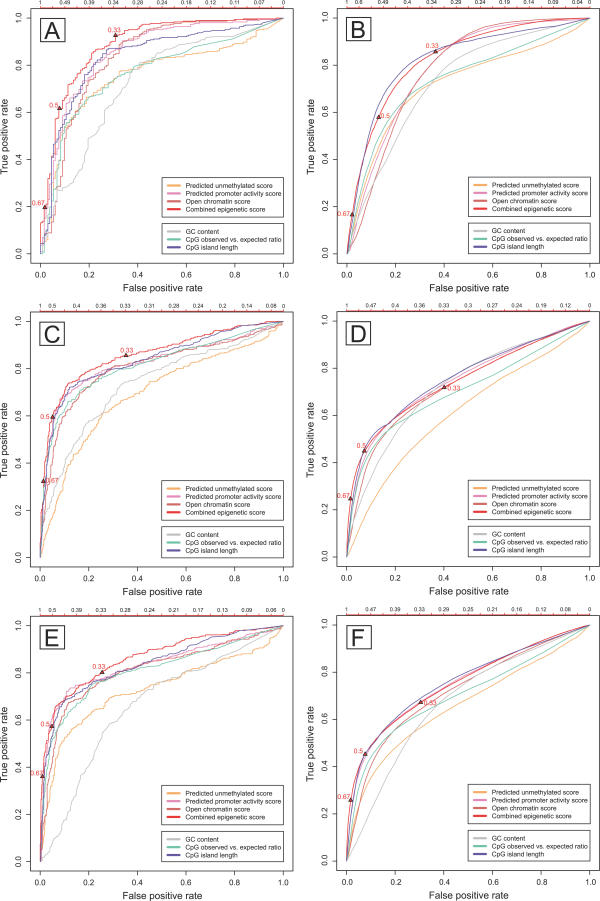
ROC Curves Comparing the Performance of Four Prediction Scores and Three Sequence Criteria against DNA Methylation and Promoter Activity This figure compares the prediction performance of four CpG island scores that are based on epigenome prediction (upper legend box) and of three simple sequence criteria (lower legend box). In (A), (C), and (E), overlap with unmethylated regions is used for evaluation, and in (B), (D), and (F), overlap with experimentally determined transcription start sites (as an indicator of promoter activity) is used instead. All graphs plot the true positive rate against the false positive rate in the form of ROC curves [27]. The scales on top of the plots display the threshold values for the combined epigenetic score that correspond to the tradeoff between false positive rate and true positive rate at any one position. The thresholds for the combined epigenetic score are highlighted by triangles: 0.5 (balance between sensitivity and specificity), 0.33 (high sensitivity), and 0.67 (high specificity). Averaged across all six graphs, the ROC area under the curve performance measure (i.e., the percentage of the unit square that lies below the ROC curve [27]) amounts to the following values: predicted unmethylated score, 65.4%; predicted promoter activity score, 74.8%; open chromatin score, 72.2%; combined epigenetic score, 75.8%, GC content, 67.1%; CpG observed-to-expected score, 70.6%; and CpG island length, 75.5%.

The ROC curves show that all four CpG island strength predictions that we constructed (i.e., the predicted unmethylated score, the predicted promoter activity score, the open chromatin score, and the combined epigenetic score) perform significantly better than random (Figure 3) and can therefore be used to improve the accuracy of CpG island mapping. Nevertheless, we observe several differences. On both evaluation datasets, the predicted unmethylated score performs worst of all four scores. This contrasts with the high accuracy of the methylation prediction itself (Table 1) and points to high divergence between the training dataset and the evaluation datasets, possibly arising from tissue specificity of DNA methylation as well as from experimental biases. The predicted promoter activity score performs well for both evaluation datasets, which is also the case for the open chromatin score. Finally, the combined epigenetic score, i.e., the consensus prediction of all three individual CpG island scores, performs better than each individual score. This result shows that the three individual scores—each derived from data for different cell types and for different aspects of CpG island strength—do provide complementary information that can be combined to increase prediction performance.

For comparison, we also plotted the performance of the GC content, the CpG observed-to-expected ratio, and the length of CpG islands, interpreting them as indicators of CpG island strength (Figure 3), and we observed a surprising result. On the one hand, GC content performs only slightly better than random, and the CpG observed-to-expected ratio—arguably the most natural sequence-based indicator of CpG island strength—performs substantially worse than the promoter activity score, the open chromatin score, and the combined epigenetic score. On the other hand, CpG island length (which one might have dismissed as a rather technical aspect of the sequence-based CpG island definition, designed to exclude short and insignificant CpG islands) turns out to perform very well, second only to the combined epigenetic score in terms of overall prediction performance (i.e., area under the ROC curve [27], averaged over Figure 3A to 3F). Although this finding contributes little to the main impetus of this paper, which is to reconcile CpG island mapping with the epigenetic and functional concept of bona fide CpG islands, it can help us to design a simple heuristic to approximate the combined epigenetic score. We discuss this point in more detail in a separate section below.

**Figure 4:**
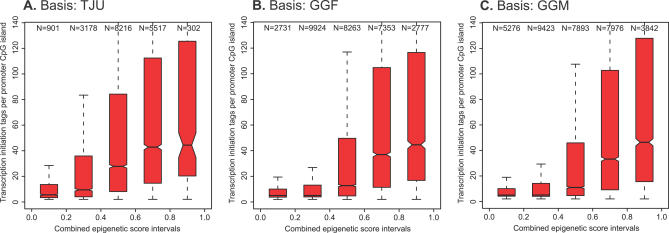
Box Plots Comparing the Promoter Strength between High-Scoring and Low-Scoring Promoter CpG Islands This figure shows box plots of the average number of transcription start site tags per CpG island (as an indicator of promoter strength), restricted to those CpG islands that show experimental evidence of promoter activity at all (i.e., at least three transcription start site tags fall within the CpG island). Separate box plots are drawn for CpG islands that fall into different intervals in terms of their combined epigenetic score (i.e., 0 to 0.2, 0.2 to 0.4, etc.). The standard box plot format is used (boxes show center quartiles, whiskers extend to the most extreme data point that is no more than 1.5 times the interquartile range from the box, and non-overlapping notches provide evidence of significantly different medians), and outliers are hidden.

In addition to the analysis by ROC curves, we performed a second evaluation, to assess whether the combined epigenetic score predicts not only the likelihood that a particular CpG island exhibits promoter activity (as shown by the ROC curves), but also the strength of its promoter activity. To that end, we plotted the number of transcription start site tags (as an indicator of promoter strength) for all CpG islands that harbor experimentally determined transcription start sites at all against the combined epigenetic score (Figure 4). The results show that promoter CpG islands with a high combined epigenetic score indeed exhibit substantially stronger promoter activity than promoter CpG islands with a low combined epigenetic score.

**Figure 5:**
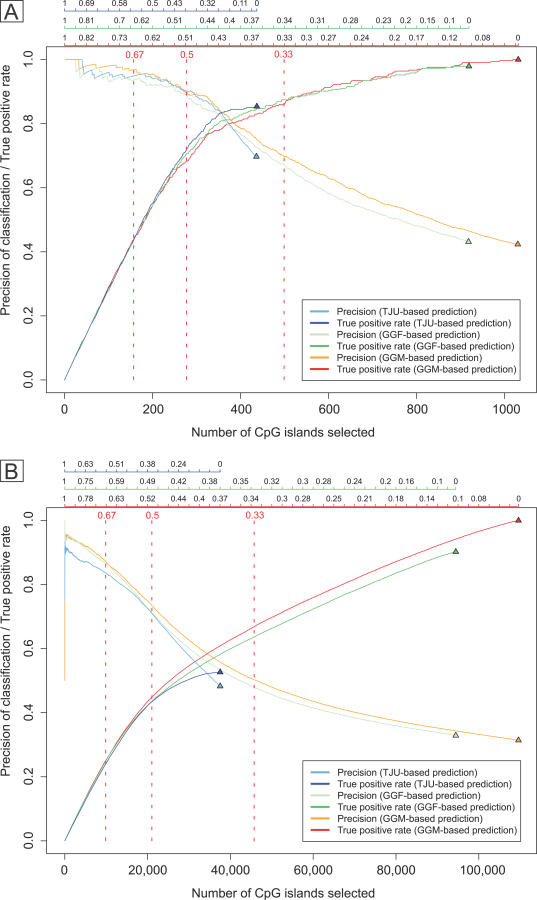
Performance of the Combined Epigenetic Score Compared between CpG Island Maps That Use Different Repeat-Exclusion Strategies This figure plots the precision (i.e., the percentage of experimentally supported bona fide CpG islands among all selected CpG islands) and the true positive rate (i.e., the percentage of experimentally supported bona fide CpG islands that are selected) over the total number of cases predicted as bona fide CpG islands, for any valid threshold on the combined epigenetic score. Evaluation criteria are absence of DNA methylation (A) and presence of promoter activity as indicated by experimentally determined transcription start sites (B). The three scales on top of each plot display the score thresholds that correspond to the number of CpG islands selected. Dashed lines show the three thresholds that were used to derive the final bona fide CpG island maps on the basis of the GGM dataset. Numbers on the *x*-axis are significantly lower in (A) than in (B) because of the fact that the DNA methylation dataset covers only a random sample of unmethylated and methylated CpG islands, while the promoter activity dataset covers essentially all nonrepetitive CpG islands genome-wide.

### Selection of the Most Appropriate CpG Island Map As the Basis for Prediction

Up to this point, we carried out all analyses in parallel for the three CpG island maps that we derived using different repeat-exclusion strategies (TJU, GGF, and GGM). To select the most appropriate setup for the final map of predicted bona fide CpG islands, we benchmarked these strategies on both evaluation datasets. Since ROC curves cannot easily account for the different number of CpG islands in each of the three maps, we constructed an alternative type of diagram for this purpose (Figure 5). This diagram plots the precision of the classification (i.e., the percentage of predicted bona fide CpG islands that are supported by the DNA methylation dataset [Figure 5A] or by the transcription start site dataset [Figure 5B]) and the true positive rate (i.e., the percentage of unmethylated CpG islands [Figure 5A] or CpG islands harboring transcription start sites [Figure 5B] that are correctly predicted) against the total number of CpG islands that are selected for any particular threshold.

The results show that there is generally high agreement between the performance of the combined epigenetic score on each of the three CpG island maps (Figure 5), apart from the trivial fact that the overall sizes of the three maps differ. Nevertheless, the combined epigenetic score performs slightly better on the GGM map (i.e., repeat exclusion using RepeatMasker, with subsequent application of the Gardiner-Garden criteria for CpG island detection) than on the two alternative maps, and this setup was therefore chosen. The GGM map has two additional advantages. First, in contrast to the GGF map, it does not require choosing a cutoff for the maximum repeat content that is permitted per CpG island. Second, in contrast to the TJU map, the DNA sequence parameters used to derive the GGM map are so permissive that virtually every nonrepetitive, CpG-rich region that exceeds 200 bp is selected and scored. Thus, scores are also calculated for regions that show little potential to be bona fide CpG islands but which may be of interest for comprehensive scans of particular genomic regions.

At http://rd.plos.org/10.1371_journal.pcbi.0030110_01, we report the combined epigenetic score for all CpG islands that fulfill the Gardiner-Garden criteria on the repeat-masked genome (GGM). Since our evaluations showed that the combined epigenetic score provides an accurate and robust estimate of CpG island strength (i.e., of a CpG island's inherent tendency to exhibit an open and transcriptionally competent chromatin structure), these scores are useful for a number of applications. For example, they add important quantitative information to support functional genome annotation as well as the interpretation of experimental epigenome data, and they can be used to prioritize candidate regions, e.g., when selecting a fixed number of most promising regulatory CpG islands for experimental followup.

### Mapping of Predicted Bona Fide CpG Islands Using the Combined Epigenetic Score

Although our analysis emphasizes the importance of quantitative information on CpG island strength, to distinguish gradually between bona fide CpG islands and those CpG-rich regions that show no evidence of a regulatory role (Figures 3 and 4), we acknowledge that certain applications would benefit from a fixed threshold on the combined epigenetic score. For example, to derive a genome-wide list of predicted bona fide CpG islands or for selecting regions to be spotted on a CpG island microarray, it is necessary to make a tradeoff between thresholds that are low enough to achieve high sensitivity (i.e., most bona fide CpG islands are included) and high enough to maintain high specificity (i.e., few CpG-rich regions that show no evidence of a regulatory role are selected).

Fortunately, the way the combined epigenetic score is defined immediately suggests a threshold that balances sensitivity and specificity and carries a biologically meaningful interpretation. Since the combined epigenetic score is the average of the confidences with which a particular CpG island is predicted (1) to be unmethylated, (2) to exhibit promoter activity, and (3) to foster open chromatin structure, it assigns a value between zero and one to each CpG island that reflects its overall epigenetic and functional state. A value of zero thus corresponds to a completely silenced, inactive, and inaccessibly buried CpG island, while a value of one corresponds to an unmethylated, highly accessible CpG island with strong promoter activity. Between these two extremes, a value of 0.5 corresponds to CpG islands that are equally likely to be bona fide CpG islands or not. This value therefore provides a suitable threshold for CpG island mapping, as it balances sensitivity and specificity. We would recommend this threshold for most applications.

Nevertheless, certain tasks (e.g., genome annotation) may require increased sensitivity to annotate as many bona fide CpG islands as possible and would therefore profit from a less stringent threshold, such as 0.33. Conversely, a highly conservative threshold of 0.67 is useful when selecting candidate regulatory regions for experimental followup, to minimize the risk of wasting resources on false positives. To support decision-making about the most appropriate map to use for a particular application, Table 4 provides quantitative data on true positive rates and false positive rates calculated for both evaluation criteria, DNA methylation and promoter activity.

**Table 4:**
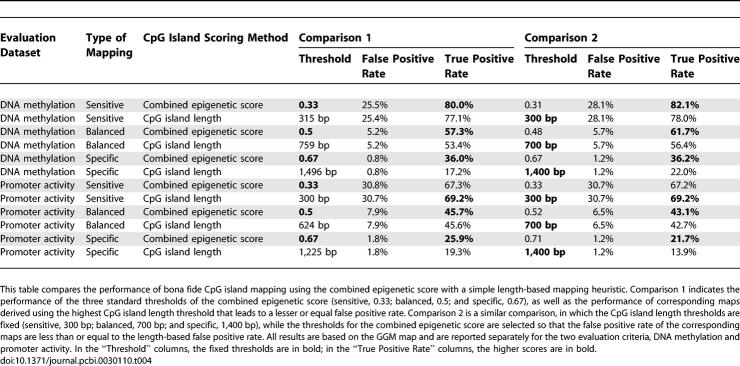
Performance Comparison between the Combined Epigenetic Score and the CpG Island Length

Using the GGM map as the basis (109,600 CpG islands for the entire human genome) and the combined epigenetic score as the indicator of CpG island strength, we calculated maps of predicted bona fide CpG islands. Using the balanced 0.5 threshold, 21,631 genomic regions are predicted as bona fide CpG islands (19.7%); for the highly sensitive 0.33 threshold, this value is 46,182 (42.1%); and for the highly specific 0.67 threshold, we predict 10,281 bona fide CpG islands genome-wide (9.4%). All CpG island maps are available for download and inspection as UCSC Genome Browser [28] tracks at http://rd.plos.org/10.1371_journal.pcbi.0030110_01.

The genomic distribution of bona fide CpG islands is summarized in Table S8. Furthermore, we assessed how frequently bona fide CpG islands associate with genes, exons, annotated transcription start sites, and highly conserved regions (Table S9). As expected, predicted bona fide CpG islands are highly associated with annotated transcription start sites and evolutionarily conserved regions, and this effect is stronger for the specific threshold than for the balanced and the sensitive thresholds. However, even of the 10,281 strongest CpG islands in the human genome, i.e., those whose scores exceed the highly specific 0.67 threshold, more than 40% do not overlap with an Ensembl-annotated transcription start site. Thus, we conclude that our prediction of CpG island strength identifies a significant number of regions with open and transcriptionally competent chromatin structure that are not known promoters of protein-coding genes.

### Evaluation of CpG Island Length As a Heuristic for the Combined Epigenetic Score

As outlined above, the combined epigenetic score has a conceptual advantage over more conventional ways of predicting CpG island strength because it directly links CpG island maps to the epigenetic and functional role that CpG islands are assumed to play in the human genome. However, it bears one significant disadvantage: the calculation of the combined epigenetic score is complex and computationally demanding. While we alleviate this issue by providing precalculated maps for the current assemblies of the human genome, it would be helpful to have a second estimate of CpG island strength available that is significantly simpler to calculate, even at the cost of a somewhat reduced performance. As suggested above and supported by Figure 3, CpG island length can be used in this way. It is substantially, though not perfectly, correlated with the combined epigenetic score (Pearson's *r* = 0.59), and it gives rise to a ROC area under the curve [27] performance that is not dramatically lower than that of the combined epigenetic score (Figure 3).

However, it is unclear what might be suitable thresholds to map bona fide CpG islands on the basis of their length, since—in contrast to the combined epigenetic score—CpG island length does not reflect any specific epigenetic concept. We propose that the most appropriate solution is to select thresholds such that the resulting maps resemble those calculated from the combined epigenetic score in terms of the false positive rate. That is, the length heuristic should not make more errors when detecting bona fide CpG islands than the combined epigenetic score, but it may well detect fewer (worse) or more (better) bona fide CpG islands, as measured by the true positive rate. Table 4 provides a performance comparison of bona fide CpG island maps derived from the combined epigenetic score versus maps derived using the CpG island length heuristic, with thresholds selected such that the false positive rate is as close as possible to that of the maps derived from the combined epigenetic score. Taking the results for both evaluation datasets into account and rounding to the closest hundred, we concluded that a minimum length of 700 bp is the most appropriate threshold for the balanced case. For sensitive mapping, the most appropriate minimum length is 300 bp, and for specific mapping, the most appropriate minimum length is 1,400 bp. Direct performance comparison with the maps derived from the combined epigenetic score shows that this length-based heuristic performs equally well for sensitive mapping (slightly worse for DNA methylation, slightly better for promoter activity), but falls short for both the balanced and the specific maps (Table 4). Differences are particularly strong for the specific case, where the map based on the combined epigenetic score predicts 65% (DNA methylation: true positive rate of 36.2% versus 22.0%) and 56% (promoter activity: 21.7% versus 13.9%) more bona fide CpG islands than the heuristic when false positive rates are fixed to 1.2% for both maps.

We conclude that the length-based heuristic can be used for a general mapping of bona fide CpG islands, preferably with a minimum length threshold of 300 bp. However, as soon as high specificity is desirable, we strongly recommend using the maps of predicted bona fide CpG islands that are based on the combined epigenetic score. This conclusion is consistent with the observation that exclusively sequence-based CpG island maps achieve high sensitivity but lack specificity, i.e., they include many regions that fail to exhibit the epigenetic and functional characteristics of bona fide CpG islands.

## Discussion

The CpG island strength as a theoretical concept captures the inherent tendency of a particular CpG island to exhibit the characteristic epigenetic and functional state of bona fide CpG islands. This includes, but is not limited to, absence of DNA methylation as well as presence and strength of promoter activity. The concept of CpG island strength is abstracted from any tissue-specific or cell-type-specific variation of the epigenetic states. It should be viewed as a description of the default state that is encoded in the DNA sequence of a particular CpG island, and which the CpG island will assume in the absence of any strong influences towards variation (such as imprinting-related differential methylation or cancer-related epigenetic silencing). Since we observed clear-cut quantitative differences among CpG islands (Figure 4) and a highly significant clustering of epigenetic modifications in a subset of CpG islands (Table 2), we conclude that this concept adds important information to traditional CpG island maps. Furthermore, it provides a straightforward solution for the lack of specificity of these maps.

To predict CpG island strength for each CpG island in the human genome, we initially predicted multiple epigenetic modifications independently. These genome-wide predictions were highly correlated with each other, hence we could combine them into a consensus prediction of CpG island strength. The predictive power of this combined epigenetic score (and of several alternative CpG island scores) was evaluated on large-scale experimental datasets of DNA methylation and promoter activity. We also selected and justified biologically plausible thresholds on the combined epigenetic score, leading to maps of predicted bona fide CpG islands that are more accurate than current sequence-based maps. For example, even the most restrictive definition [16] of CpG islands (TJU) gives rise to approximately one-third methylated CpG islands, i.e., CpG-rich regions that fail to exhibit the characteristics of bona fide CpG islands according to our evaluation dataset. Using a sensitive threshold of 0.33 on the combined epigenetic score, this value can be reduced by two-thirds, while losing less than 8% of unmethylated, potentially bona fide CpG islands (Figure 3A). Similar improvements were observed when evaluating promoter activity and for two additional CpG island maps (GGF and GGM). We therefore conclude that a post-processing step utilizing bioinformatic predictions significantly increases the accuracy of CpG island mapping and can help overcome the weaknesses of current CpG island definitions. We also showed that a simple length-based mapping heuristic that selects only CpG islands with a minimum length of 300 bp on the repeat-masked genome is suitable for sensitive mapping of bona fide CpG islands but performs substantially worse than the combined epigenetic score when high specificity is desired.

The fundamental advance of our analysis was to move beyond a purely sequence-based definition of CpG islands (which many researchers have tried to optimize in the past [29–33]) and to incorporate epigenome and chromatin data. This approach is consistent with the common notion of CpG islands being functionally and epigenetically exceptional regions, but gave rise to two conceptual difficulties. First, such data are tissue-specific and cell-type-specific. It is thus necessary to abstract the experimental data from these variations to derive a single CpG island map for the human genome (instead of specific maps for all major tissues and cell types). Second, comprehensive epigenome data are currently available only for Chromosomes 21 and 22, not for the entire genome. We addressed both issues by introducing epigenome prediction as the method for scoring CpG island strength, instead of using epigenome data directly.

**Figure 6:**
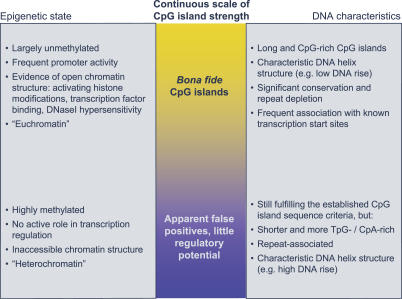
Parallelism between Specific DNA Characteristics and the Epigenetic and Functional State of CpG Islands This figure illustrates the link between the genome sequence and the epigenome at CpG islands, which enabled us to predict epigenetic states from characteristics of the genome sequence. CpG islands in the human genome can apparently be ordered on a scale of increasingly open and transcriptionally competent chromatin structure (left) and simultaneously on a scale of characteristic DNA attributes (right), with high correlation between both scales.

Our epigenome predictions utilize a strong link that connects the DNA characteristics of individual CpG islands with their epigenetic states. As illustrated schematically in Figure 6, CpG islands differ in terms of their epigenetic states, in particular in their inherent tendency towards either open and transcriptionally competent or inaccessible and silenced chromatin structure. Similarly, CpG islands differ in terms of their DNA characteristics and genomic locations, including length and CpG frequency, preferred DNA helix structure, association with conserved regions, frequency of transcription start sites, and distribution of repetitive DNA elements. Intriguingly, epigenetic state and DNA characteristics are highly correlated, as indicated by the consistently high prediction accuracies that we observed throughout this study: CpG islands that are frequently unmethylated, exhibit promoter activity, and/or foster open chromatin structure also exhibit exceptional DNA characteristics, including high levels of CpG enrichment, high conservation, significant repeat depletion, and a specific predicted helix structure. On the other hand, methylated and transcriptionally inactive regions (that still fulfill the traditional CpG island criteria) exhibit converse DNA characteristics. This high degree of correlation between DNA characteristics and epigenetic state extends beyond CpG islands: our prediction pipeline also achieved high prediction performances for the distinction between regions that exhibit an open and transcriptionally competent chromatin structure and a set of randomly selected genomic regions (unpublished data). We therefore conclude that the human genome and epigenome are significantly correlated.

Potential limitations of this study arise from the epigenome datasets that were employed for training and evaluation. First, two out of the five ChIP-on-chip datasets that we used are based on ligation-mediated PCR amplification [9,12], which creates an experimental bias towards GC-rich regions (the other three are based on a more appropriate linear DNA amplification method [10]). Second, the lists of over-represented regions from the ChIP-on-chip studies that we used are most likely overly conservative [34]. However, in spite of these shortcomings of the underlying datasets, we observed consistent results across multiple datasets, which were obtained from different cell types, in different labs, and with different experimental protocols. Therefore, such error sources are highly unlikely to invalidate our main results. A second limitation concerns our ability to exhaustively evaluate the performance of the predictions: because the concepts of CpG island strength and of bona fide CpG islands describe inherent properties of CpG islands, which abstract from their epigenetic state in a particular tissue or cell type, they are difficult to measure experimentally. We therefore performed our evaluations on datasets that significantly deviate in their experimental and biological characteristics from all training data that was used, and we paid as much attention to deriving consistent and biologically plausible predictions of CpG island strength as to achieving the highest performance on the evaluation criteria. Finally, for reasons of data availability we focused on epigenetic modifications that are associated with open and transcriptionally competent chromatin. Future extensions of this work should include repressive epigenetic modifications as well, such as histone H3K9 methylation and H3K27 methylation. On this basis, combined with larger datasets, it may be possible to deconstruct the predicted CpG island strength into individual components for all major epigenetic modifications.

The CpG island strength predictions and maps of predicted bona fide CpG islands described in this study are currently being used in several ongoing research projects, with topics ranging from imprinting regulation and epigenome profiling [35] to cancer-related hypermethylation, and have so far proved to be highly useful, both for guiding the selection of candidate regulatory regions and for supporting the interpretation of experimental results.

## Materials and Methods

### CpG island maps.

To calculate genome-wide CpG island maps according to the traditional sequence-based definition, we downloaded both the unmasked and the repeat-masked versions of the hg17/NCBI35 human genome assembly from the UCSC Genome Browser Web site [28], and we ran a slightly modified version of the CpG Island Searcher script [16] with the following parameters. Calculation of the TJU map: GC content above 55%, CpG observed-to-expected ratio above 0.65, length above 500 bp, based on the unmasked genome. Calculation of the GGF map: GC content above 50%, CpG observed-to-expected ratio above 0.6, length above 200 bp, based on the unmasked genome. Calculation of the GGM map: GC content above 50%, CpG observed-to-expected ratio above 0.6, length above 200 bp, based on the repeat-masked genome. Finally, for GGF we determined the number of nonrepetitive basepairs by comparison with the repeat-masked genome version and discarded all CpG islands for which this value was below 200 bp.

### Epigenome prediction pipeline.

For the prediction of DNA methylation, promoter activity, and the five components of the open chromatin score, we implemented a custom computer program. This epigenome prediction pipeline is based on our experience with the prediction of DNA methylation published previously [20], and it implements several significant extensions. First, a 20-fold speedup of the program over the original version, achieved by optimization of the source code and of the database structure, now permits genome-wide analysis at acceptable speed. Second, a front end for Web-based analysis was implemented, which enables us to make the prediction pipeline available to interested researchers on a cooperation basis (see http://rd.plos.org/10.1371_journal.pcbi.0030110_02 for details). Unrestricted public access to this Web service is not yet feasible because of high computational demand of the prediction pipeline, but it is planned for the future.

Briefly, the prediction pipeline works as follows. It takes a training set as input that consists of two lists of genomic positions (i.e., chromosome, start and end of the region relative to the hg17/NCBI35 genome assembly), the first one representing the positive cases and the second one the negative cases. Then, four consecutive steps are performed.

First, to prepare the DNA-based prediction, a wide range of DNA attributes are calculated for all training cases and, in addition, for all CpG islands in the human genome. These attributes belong to six classes: (1) DNA sequence patterns and properties (426 attributes), (2) repeat attributes, frequency, and distribution (311 attributes), (3) predicted DNA helix structure (28 attributes), (4) predicted transcription factor binding sites (68 attributes), (5) evolutionary conservation and single nucleotide polymorphisms (ten attributes), and (6) CpG island attributes (four attributes). Most attributes take the form of frequencies or numerical scores, averaged over the CpG island and standardized to a default window size of one kilobase (see Table S10 for the full list of attributes and for information on their calculation). The data for most of these attributes were collected from annotation tracks in the UCSC Genome Browser [28] (as of September 2005), with some exceptions: the attributes for classes 1 and 6 were calculated directly from the DNA sequence, and the attributes for class 3 were calculated from the DNA sequence by averaging over oligonucleotides with known structure [24].

Second, to estimate the prediction performance that a linear support vector machine can achieve for classification of positives and negatives, it is repeatedly trained and tested on partitions of the training dataset following a four-step procedure. (1) If the larger set (either positives or negatives) contains more than twice as many sites as the smaller set, it is randomly downsampled such that the class imbalance never exceeds 67% versus 33% (this precaution limits potential bias towards predicting the majority class). (2) Using 10-fold cross-validation, a linear support vector machine [36] as implemented in the Weka package [37] is repeatedly trained on 90% of the cases and tested on the remaining 10% (with default parameters). (3) Cross-validation is repeated ten times with random partition assignments. (4) The overall prediction performance is measured by the correlation coefficient between the predictions and the correct values on the test set of the cross-validations and by the percent accuracy of correctly predicted test set cases [38], averaged over all cross-validation runs.

Third, to understand which DNA attributes contribute to high prediction performances, the analysis described in the previous step is repeated for all six attribute groups separately (Tables S1, S2, and S5). In addition, single-attribute significance testing is performed on all 847 attributes (Tables S3, S4, and S6), using the nonparametric Wilcoxon rank-sum test with an overall significance threshold of 5% per statistical analysis. *p*-Values are adjusted for multiple testing alternatively by the highly conservative Bonferroni method (which controls the family-wise error rate) and by a more recent method that controls the false discovery rate [39].

Fourth, to derive a score for all CpG islands in the human genome, a linear support vector machine is trained as described above, but now on the full training dataset (with downsampling if necessary, to enforce a maximum class imbalance of 67% versus 33%). The trained prediction model is then used to predict the likelihood of belonging to the set of positives for each CpG island genome-wide. Likelihoods are calculated as implemented by the Weka package [37]. The resulting quantitative predictions can assume values between zero and one, where a value of zero corresponds to a high-confidence negative prediction, a value of 0.5 to a borderline case, and a value of one to a high-confidence positive prediction. This quantitative prediction can then be used directly as a CpG island score or it can be subjected to further calculations as described below.

### Prediction scores for CpG island strength.

The calculation of all four CpG island scores made use of the prediction pipeline, combined with appropriate training data. Calculations were performed on the hg17/NCBI35 genome assembly. Where necessary, data were remapped using the UCSC Genome Browser liftOver tool [28].

The predicted unmethylated score is based on training data from an experimental analysis of CpG island methylation in human lymphocytes [18] (dataset obtained from the supplementary material of [18]). Using methylation-specific restriction enzyme and PCR, Yamada et al. measured DNA methylation states for 149 CpG-rich regions on Chromosome 21q, of which 132 cases showed an unambiguous methylation pattern and could be mapped to the current genome assembly. All CpG islands that overlap (by at least 1 bp) with one of the 103 unmethylated regions were combined into the positive training set, and all CpG islands that overlap with one of the 29 methylated cases were combined into the negative training set. The resulting training dataset was then processed by the prediction pipeline to derive predicted unmethylated scores for all CpG islands according to TJU, GGF, and GGM.

The predicted promoter activity score is based on training data from an experimental analysis of polymerase II preinitiation complex binding in human fibroblasts [9] (dataset obtained from the supplementary material of [9]). Using the ChIP-on-chip protocol and a highly conservative method for identifying regions of over-representation from the raw data, Kim et al. derived a genome-wide map of the most likely binding sites. All CpG islands on Chromosome 21 and 22 that overlap by at least 1 bp with one of these binding sites were combined into the positive training set. The negative training set was constructed from those CpG islands on Chromosome 21 and 22 that are at least 500 bp away from the nearest binding site. The resulting training dataset was then processed by the prediction pipeline to derive predicted promoter activity scores for all CpG islands according to TJU, GGF, and GGM.

The open chromatin score is based on training data from several large-scale analyses. (1) Using the ChIP-on-chip protocol, Bernstein et al. [10] derived histone modification data for the HepG2 cell line, including H3K4 di- and trimethylation and H3K9/14 acetyla-tion (dataset obtained from http://www.broad.mit.edu/cell/chromatin_study). Their analysis comprised the nonrepetitive parts of Chromosomes 21 and 22, for which they calculated sites of significant over-representation. (2) Using DNase I digestion and subsequent massively parallel signature sequencing, Crawford et al. [13] derived a genome-wide profile of DNase I hypersensitive sites in CD4+ T cells (dataset obtained from the UCSC Genome Browser [28]). (3) Using the ChIP-on-chip protocol, Cawley et al. [12] derived binding data for the ubiquitous transcription factor SP1 in the Jurkat cell line (dataset obtained from http://transcriptome.affymetrix.com/publication/tfbs). Their data comprises the nonrepetitive parts of Chromosomes 21 and 22, for which they calculated sites of significant over-representation. For each of the five epigenetic modifications, respectively, we constructed a training dataset as follows. All CpG islands on Chromosome 21 and 22 that overlap with the most significant sites for the respective epigenetic modification (as reported by the original authors) were included in the positive training set, and all CpG islands on Chromosome 21 and 22 that were at least 500 bp away from the nearest site were included in the negative training set. All five resulting training datasets were then processed by the prediction pipeline, and the five predictions for each CpG island were averaged, to derive open chromatin scores for all CpG islands according to TJU, GGF, and GGM.

The combined epigenetic prediction score is calculated for each CpG island as the (unweighted) average of its predicted unmethylated score, its predicted promoter activity score, and its open chromatin score. Since all three components can assume values from zero to one, the same is true for their average.

### Evaluation on experimental datasets of DNA methylation and promoter activity.

For the evaluation on DNA methylation, we used a dataset by Rollins et al. [25], who identified 3,073 unmethylated and 2,565 methylated domains in human brain tissue (dataset obtained from http://epigenomics.cu-genome.org/html/meth_landscape). Their data are based on paired-end sequencing from two DNA libraries that were constructed by digestion with methylation-sensitive restriction enzymes, such that one library is highly enriched with unmethylated regions while the other contains almost exclusively methylated regions. We regarded a CpG island as unmethylated if it overlapped by at least 25% with an unmethylated domain and as methylated if it overlapped by at least 25% with a methylated domain. No cases were observed where a single CpG island overlapped with an unmethylated and a methylated domain simultaneously.

For the evaluation of promoter activity, we used a dataset from the FANTOM3 consortium [26], who performed large-scale CAGE analysis (i.e., tag sequencing of 5′ ends of full-length mRNA) on cDNA libraries derived from a wide range of tissues and cell types (dataset obtained from http://gerg01.gsc.riken.jp/cage_analysis/export/hg17prmtr). All CpG islands that contained at least three tags (i.e., experimental evidences of independent transcription initiation events) were regarded as CpG islands with promoter activity, while all other cases were regarded as CpG islands that show either no or only spurious promoter activity.

ROC curves were constructed in the usual way [27], using the ROCR library [40] and the R statistical package (http://www.r-project.org). The diagrams that compare the different repeat-exclusion strategies (Figure 5) were constructed using the same tools, with some customizing to ensure that every unmethylated domain is counted only once for the true positive rate, even if it overlaps with several CpG islands simultaneously. All R scripts are available on request.

### Co-localization analysis.

To show that the five components of the open chromatin score exhibit significant overlap with each other and with the three CpG island maps (TJU, GGF, and GGM), we performed a co-localization analysis of these eight datasets on Chromosomes 21 and 22. To this end, a custom script was written that counts the number of sites of one type that overlap with a second type, for all pairs of site types (i.e., epigenetically modified regions and CpG islands). From these values, overlap percentages were calculated and plotted as a heat map (Figure 2A).

However, frequent and long regions are obviously more likely to overlap with other sites than are rare and short regions. We therefore normalized the observed frequency of overlap by the expected frequency for a uniform distribution, using the following procedure. (1) For each site type, we derived a random control set with similar set size, length distribution, and repeat overlap. Technically, for each record in the corresponding dataset, a random site of identical length was drawn from the entire length of Chromosomes 21 and 22. If this random site was within five percentage points of its corresponding record in terms of repeat content, it was retained; otherwise, a new random site was drawn. (2) Pairwise frequencies of overlap between all control regions were counted. (3) Steps 1 and 2 were repeated 20 times, and frequencies of overlap were averaged. (4) The observed frequencies of overlap for the real data were divided by the averaged random overlap frequencies, giving rise to *n*-fold over- and under-representations. Figure 2B reports base-2 log scores of these over-representations (under-representation relative to the expected overlap did not occur).

### Data availability.

Genome-wide maps of predicted bona fide CpG islands and CpG island strength scores can be accessed online and downloaded at http://rd.plos.org/10.1371_journal.pcbi.0030110_01. Furthermore, they are available as custom tracks on the UCSC Genome Browser Web site and as Distributed Annotation System tracks (www.biodas.org) for visualization within the Ensembl genome browser. The source code of the prediction pipeline is available on request from cbock@mpi-inf.mpg.de.

## Supporting Information

Table S1 Prediction Performances for the Distinction between Unmethylated and Methylated CpG Islands (19 KB XLS)

Table S2 Prediction Performances for the Distinction between CpG Islands That Show Evidence of Promoter Activity and Those That Do Not (19 KB XLS)

Table S3 List of Significantly Different DNA Attributes between Unmethylated and Methylated CpG Islands (34 KB XLS)

Table S4 List of Significantly Different DNA Attributes between CpG Islands That Show Evidence of Promoter Activity and Those That Do Not (34 KB XLS)

Table S5 Prediction Performances for the Distinction between CpG Islands That Show Evidence of Open Chromatin and Those That Do Not (34 KB XLS)

Table S6 List of Significantly Different DNA Attributes between CpG Islands That Show Evidence of Open Chromatin and Those That Do Not (112 KB XLS)

Table S7 Correlation among the Epigenome Predictions That Contribute to the Open Chromatin Score (23 KB PDF)

Table S8 Distribution of Bona Fide CpG Islands along the Human Chromosomes (23 KB XLS)

Table S9 Association of Bona Fide CpG Islands with Genes and Evolutionary Conserved Regions (18 KB XLS)

Table S10 Overview of the DNA Attributes Used for Prediction and Statistical Analysis (66 KB DOC)

## Abbreviations

bp: basepair(s)
GGF: Gardiner-Garden filtered
GGM: Gardiner-Garden masked
TJU: Takai Jones unmasked
ROC: receiver operating characteristic
