# Supplementary material for: CpG Island Mapping by Epigenome Prediction
Source: PLoS Comput Biol. 2007 Jun 8;3(6):e110. doi: 10.1371/journal.pcbi.0030110 (PMC1892605; doi:10.1371/journal.pcbi.0030110)
Supplement: Supplementary file 1 [file pcbi.0030110.st007.pdf]

Supplementary Table 7 – Correlation among the epigenome predictions that contribute to the open chromatin score

A. Correlation among epigenome predictions at CpG islands, TJU-based

Correlations

		Predicted overlap with H3K4Me2	Predicted overlap with H3K4Me3	Predicted overlap with H3K9ac / H3K14ac	Predicted overlap with DNaseI hypers.	Predicted overlap with Sp1 binding	Open CpG island score
Predicted overlap with H3K4Me2	Pearson Correlation	1	.596**	.599**	.566**	.448**	.772**
	Sig. (2-tailed)		.000	.000	.000	.000	.000
	N	37531	37531	37531	37531	37531	37531
Predicted overlap with H3K4Me3	Pearson Correlation	.596**	1	.890**	.602**	.572**	.874**
	Sig. (2-tailed)	.000		.000	.000	.000	.000
	N	37531	37531	37531	37531	37531	37531
Predicted overlap with H3K9ac / H3K14ac	Pearson Correlation	.599**	.890**	1	.601**	.553**	.870**
	Sig. (2-tailed)	.000	.000		.000	.000	.000
	N	37531	37531	37531	37531	37531	37531
Predicted overlap with DNaseI hypersensitivity	Pearson Correlation	.566**	.602**	.601**	1	.711**	.836**
	Sig. (2-tailed)	.000	.000	.000		.000	.000
	N	37531	37531	37531	37531	37531	37531
Predicted overlap with Sp1 binding	Pearson Correlation	.448**	.572**	.553**	.711**	1	.803**
	Sig. (2-tailed)	.000	.000	.000	.000		.000
	N	37531	37531	37531	37531	37531	37531
Open CpG island score	Pearson Correlation	.772**	.874**	.870**	.836**	.803**	1
	Sig. (2-tailed)	.000	.000	.000	.000	.000	
	N	37531	37531	37531	37531	37531	37531

**. Correlation is significant at the 0.01 level (2-tailed).

B. Correlation among epigenome predictions at CpG islands, GGF-based

Correlations

		Predicted overlap with H3K4Me2	Predicted overlap with H3K4Me3	Predicted overlap with H3K9ac / H3K14ac	Predicted overlap with DNaseI hypers.	Predicted overlap with Sp1 binding	Open CpG island score
Predicted overlap with H3K4Me2	Pearson Correlation	1	.842**	.791**	.764**	.607**	.909**
	Sig. (2-tailed)		.000	.000	.000	.000	.000
	N	94450	94450	94450	94450	94450	94450
Predicted overlap with H3K4Me3	Pearson Correlation	.842**	1	.849**	.715**	.623**	.916**
	Sig. (2-tailed)	.000		.000	.000	.000	.000
	N	94450	94450	94450	94450	94450	94450
Predicted overlap with H3K9ac / H3K14ac	Pearson Correlation	.791**	.849**	1	.741**	.663**	.921**
	Sig. (2-tailed)	.000	.000		.000	.000	.000
	N	94450	94450	94450	94450	94450	94450
Predicted overlap with DNaseI hypersensitivity	Pearson Correlation	.764**	.715**	.741**	1	.521**	.855**
	Sig. (2-tailed)	.000	.000	.000		.000	.000
	N	94450	94450	94450	94450	94450	94450
Predicted overlap with Sp1 binding	Pearson Correlation	.607**	.623**	.663**	.521**	1	.784**
	Sig. (2-tailed)	.000	.000	.000	.000		.000
	N	94450	94450	94450	94450	94450	94450
Open CpG island score	Pearson Correlation	.909**	.916**	.921**	.855**	.784**	1
	Sig. (2-tailed)	.000	.000	.000	.000	.000	
	N	94450	94450	94450	94450	94450	94450

**. Correlation is significant at the 0.01 level (2-tailed).

C. Correlation among epigenome predictions at CpG islands, GGM-based

Correlations

		Predicted overlap with H3K4Me2	Predicted overlap with H3K4Me3	Predicted overlap with H3K9ac / H3K14ac	Predicted overlap with DNaseI hypers.	Predicted overlap with Sp1 binding	Open CpG island score
Predicted overlap with H3K4Me2	Pearson Correlation	1	.757**	.747**	.719**	.541**	.838**
	Sig. (2-tailed)		.000	.000	.000	.000	.000
	N	109600	109600	109600	109600	109600	109600
Predicted overlap with H3K4Me3	Pearson Correlation	.757**	1	.925**	.754**	.730**	.947**
	Sig. (2-tailed)	.000		.000	.000	.000	.000
	N	109600	109600	109600	109600	109600	109600
Predicted overlap with H3K9ac / H3K14ac	Pearson Correlation	.747**	.925**	1	.742**	.703**	.933**
	Sig. (2-tailed)	.000	.000		.000	.000	.000
	N	109600	109600	109600	109600	109600	109600
Predicted overlap with DNaseI hypersensitivity	Pearson Correlation	.719**	.754**	.742**	1	.563**	.857**
	Sig. (2-tailed)	.000	.000	.000		.000	.000
	N	109600	109600	109600	109600	109600	109600
Predicted overlap with Sp1 binding	Pearson Correlation	.541**	.730**	.703**	.563**	1	.822**
	Sig. (2-tailed)	.000	.000	.000	.000		.000
	N	109600	109600	109600	109600	109600	109600
Open CpG island score	Pearson Correlation	.838**	.947**	.933**	.857**	.822**	1
	Sig. (2-tailed)	.000	.000	.000	.000	.000	
	N	109600	109600	109600	109600	109600	109600

**. Correlation is significant at the 0.01 level (2-tailed).
