# Supplementary material for: CpG Island Mapping by Epigenome Prediction
Source: PLoS Comput Biol. 2007 Jun 8;3(6):e110. doi: 10.1371/journal.pcbi.0030110 (PMC1892605; doi:10.1371/journal.pcbi.0030110)
Supplement: Supplementary file 2 [file pcbi.0030110.st010.doc]

### Supplementary Information 10 – Overview of DNA attributes used for prediction and statistical analysis

*General remarks*

In order to make the attributes comparable between multiple regions of different sizes, most attributes are either aggregated over the region (mean, standard deviations, etc.) or standardized to a 1000 basepair window (e.g. for overlap lengths).

Because many attributes share similar calculation patterns, we give an overview of typical calculations here, to which we will refer many times below.

*Distributions*:

_avg	=	mean

_std	=	standard deviation

_skew	=	skewness

_kurt	=	kurtosis

*Patches*:

Only feature patches are counted where the feature overlaps the window by at least 25% of the feature or 10% of the window. From the set of valid patches, the following attributes are calculated:

_len	=	total length of overlap with window, standardized to 1000 basepairs

_no	=	total number of patches, standardized to 1000 basepairs

_avg	=	average of full lengths, not only the overlap

_std	=	standard deviation of full lengths, not only the overlap

_sco	=	[only for *scored patches*] mean score (no weighting by length)

(i) *DNA sequence patterns and properties*

| **Attribute Name(s)** | **Description** | **Implementation / Reference** |
| --- | --- | --- |
| Pat_AAAA to  Pat_TTTT | Pattern frequency (strand-specific, +-strand only) of all 4-mers | By simple counting on the +-strand of the genomic sequence (NCBI35) |
| Uni_AAAA  To Uni_CTTT | Pattern frequency (not strand-specific, both strands) of all 4-mers | By simple counting on the +-strand and saving to either the pattern or the reverse complement, whichever comes earlier in the alphabet |
| P_TATATA etc. | Pattern frequency (strand-specific, +-strand only) of a few arbitrarily picked special purpose patterns | By simple counting on the +-strand of the genomic sequence (NCBI35) |
| fG_avg  fG_std  fG_skew  fG_kurt fC_avg  fC_std  fC_skew  fC_kurt  fCG_avg  fCG_std  fCG_skew  fCG_kurt  fOE_avg  fOE_std  fOE_skew  fOE_kurt | Frequency distribution of Cs, Gs, CpGs, and of the Observed / Expected ratio | Calculated directly from sequence (NCBI35), over non-overlapping 50bp windows. Average, std-dev, skewness, and kurtosis are then calculated for the resulting distribution |

(ii) *Repeat attributes, frequency and distribution*

| **Attribute Name(s)** | **Description** | **Implementation / Reference** |
| --- | --- | --- |
| WDu_len  WDu_no  WDu_avg  WDu_std | Confirmed segmental duplications, defined as having similarity to sequences in the Segmental Duplication Database (SDD) | From UCSC Genome Browser “WSSD Duplication Track”: celeraDupPositive.ChromStart and ChromEnd in the usual way for patches |
| SDu_len  SDu_no  SDu_avg  SDu_std  SDu_sco | Duplications of at least 1000 basepairs of the total sequence (containing at least 500 bp of non-RepeatMasked sequence) with a sequence identity of at least 90% | From UCSC Genome Browser “Segmental Duplications”: genomicSuperDups.ChromStart and ChromEnd in the usual way for scored patches |
| SAl_len  SAl_no  SAl_avg  SAl_std  SAl_sco | Alignments of the human genome with itself, using a gap scoring system that allows longer gaps than traditional affine gap scoring systems | From UCSC Genome Browser “Self Chain”: chr<No>_chainSelf.ChromStart and ChromEnd in the usual way for scored patches |
| Rep_len  Rep_no  Rep_avg  Rep_std  Rep_sco  Rep_ssco  Rep_sta  Rep_ssta  Rep_end  Rep_send  Rep_lef  Rep_slef  RCl_XXXX  RCn_XXXX  RCa_XXXX  RCs_XXXX  RC1_XXXX  RC2_XXXX  RC3_XXXX  RC4_XXXX  RC5_XXXX  RC6_XXXX  RC7_XXXX  RC8_XXXX  RFl_YYYY  RFn_YYYY  RFa_YYYY  RFs_YYYY  RF1_YYYY  RF3_YYYY  RF5_YYYY  RF7_YYYY | Repeats found by RepeatMasker.  XXXX stands for repeat classes (SINE, LINE, etc.), and YYYY for repeat families (L1, L2, Alu, etc.)  Rep_sco = mean of Smith-Waterman alignment score (1)  Rep_ssco = std. dev. (2)  Rep_sta = alignment start relative to full repeat (3)  Rep_ssta = std. dev. (4)  Rep_end = alignment end relative to full repeat (5)  Rep_send = std. dev. (6)  Rep_lef = no. of bases of full repeat that are unaligned (7)  Rep_slef = std. dev. (8)  (for classes: mean and stddev; for families: only mean) | From UCSC Genome Browser “RepeatMasker”: chr<No>_rmsk in the usual way for scored patches |
| Tan_len  Tan_no  Tan_avg  Tan_std  Tan_per  Tan_sper  Tan_cop  Tan_scop  Tan_sco  Tan_ssco  Tan_ent  Tan_sent | Simple tandem repeats (possibly imperfect) located by Tandem Repeats Finder  Period, mean + std. dev  Copy number, mean + std. dev  Score, mean + std. dev  Entropy, mean + std. dev | From UCSC Genome Browser “Simple Repeats”: simpleRepeat in the usual way for scored patches |

(iii) *Predicted DNA helix structure*

| **Attribute Name(s)** | **Description** | **Implementation / Reference** |
| --- | --- | --- |
| SASA_avg  SASA _std  SASA _skew  SASA _kurt | Solvent accessible surface area of the DNA, predicted from sequence using oligomers with known structure | Prediction for each base from the DNA sequence according to J. Greenbaum’s solvent accessibility prediction, based on “22_Solvent_ Accessibility_Trimers.txt” (J. Greenbaum. |
| twis_avg  twis_std  twis_skew  twis_kurt  roll_avg  roll_std  roll_skew  roll_kurt  tilt_avg  tilt_std  tilt_skew  tilt_kurt  rise_avg  rise_std  rise_skew  rise_kurt  slid_avg  slid_std  slid_skew  slid_kurt  shif_avg  shif_std  shif_skew  shif_kurt | Basic structural properties of the DNA, predicted from sequence using oligomers with known structure | Prediction for each base from the DNA sequence based on “23_Octamer_Structure_ Attributes.txt”, averaging over all eight overlapping octamers |

(iv) *Predicted transcription factor binding sites:*

| **Attribute Name(s)** | **Description** | **Implementation / Reference** |
| --- | --- | --- |
| TF_Total  TF_XXX | Number of human/mouse/rat conserved, computationally identified, transcription factor binding sites in window (XXX stands for any transcription factor with a known binding matrix – all TFs starting with the same three letters are assumed to form a group and are counted together) | From UCSC Genome Browser “TFBS Conserved”: tfbsConsSites by simple counting (after manual removal of TFBS that have a frequency of less than 5 on chrom21q). To get some aggregation, TF where the first three letters are identical are put into the same category |

(v) *Evolutionary conservation and single nucleotide polymorphisms*

| **Attribute Name(s)** | **Description** | **Implementation / Reference** |
| --- | --- | --- |
| Mos_len  Mos_no  Mos_avg  Mos_std  Mos_sco  Mos_ssco | Elements of high conservation as calculated by PhastCons over pairwise Human/Chimp/Mouse/ Rat/Dog/Chick/Fugu/Zfish  whole genome alignments | From UCSC Genome Browser “Most Conserved”: phastConsElements in the usual way for scored patches |
| Con_avg  Con_std  Con_skew  Con_kurt | Conservation score distribution | From UCSC Genome Browser “Conservation Track”:  Calculate average, std-dev, skewness, and kurtosis of the 1 bp resolution distribution in window |
| S_count  SC_XXXXX  SF_YYYYY | Total number of SNPs in window, number of SNP by class (snp, in-del, …), and by function (manually simplified) | From UCSC Genome Browser “SNPs”: snp by simple counting and use of the first 5 characters of ‘class’ as XXXXX and of ‘func’ as YYYYY |

(vi) *CpG island attributes*

| **Attribute Name(s)** | **Description** | **Implementation / Reference** |
| --- | --- | --- |
| fG_avg  fC_avg  fOE_avg  Length | Window characteristics: G content, C content, CpG observed vs. expected ratio, and window length | Calculated directly from the genome sequence.  G and C content are measured separately in order to enable the detection of “mathematical CpG islands” (Takai / Jones 2002). |
